# Evaluation the impact of electricity consumption on China’s air pollution at the provincial level

**DOI:** 10.1371/journal.pone.0301537

**Published:** 2024-04-16

**Authors:** Shen Zhong, Yu’an Fang, Hongjun Jing

**Affiliations:** 1 School of Finance, Harbin University of Commerce, Harbin City, Heilongjiang Province, China; 2 School of Public Finance and Administration, Harbin University of Commerce, Harbin City, Heilongjiang Province, China; National University of Sciences and Technology, PAKISTAN

## Abstract

As the world’s largest electricity-consuming country, China faces the challenge of energy conservation and environmental pollution. Therefore, it is imperative that China takes decisive action to address these issues. Based on the panel data of 30 provinces (cities, districts) in China from 2011 to 2020, we use the entropy method to measure the air pollution index in different provinces, construct two fixed effects models, panel quantile model, and spatial Durbin model to empirically analyze the impact of electricity consumption on air pollution in China’s provincial regions. The experimental results show that: (1) Electricity consumption has a significant positive impact on the provincial air pollution index in China and the higher the index is, the more serious the air pollution is. When the electricity consumption increases 1%, the air pollution index will increase of by 0.0649% as accompanied. (2) Through comparison of different times, we found that the degree of increase in air pollution index caused by electricity consumption would be reduced due to the improvement of environmental protection efforts. From the perspective of different geographical locations, the electricity consumption in the southeast side of the "Hu Line" has exacerbated the impact on air pollution index. (3) According to the panel quantile regression results, the marginal effect of electricity consumption on air pollution is positive. With the increase of quantiles, the impact of electricity consumption on air pollution is increasing. (4) Spatial effect analysis shows that electricity consumption has a significant positive spatial spillover effect on air pollution index. The increase in electricity consumption not only increases the air pollution index in the local region, but also leads to an increase in the air pollution index in surrounding areas. These findings contribute to the governance of air pollution and the promotion of sustainable economic, environmental and energy development.

## 1. Introduction

Since 1978, China’s per capita GDP has increased from $330 in 1978 to breaking through $10,000 in 2019 and then surpassing $12,000 in 2022. However, the rapid economic development has also brought a series of social problems. Among them, environmental pollution, especially air pollution, is an important problem that needs to be solved urgently [1]. According to the 2022 EPI report, China’s overall environmental performance ranks 160th among 180 countries, while its air quality assessed by environmental particulate matter and other indicators also ranks last. Based on the 2022 China Environmental Health Report, it was discovered that out of the 339 cities in China, 126 cities have exceeded the standards for air pollution, accounting for over 30%. Although there has been a significant improvement in air pollution levels in most cities in recent years, it was discovered that only Lhasa’s air pollution level is lower than the annual average safety value set by the World Health Organization (WHO) for PM2.5 data. In 2019, China experienced a staggering death toll of 1.85 million people due to air pollution-related issues, with 1.42 million deaths being attributed to particulate matter in the air [2]. At the same time, existing studies have found that air pollution has had an important impact on China’s economic and social development [3].

At present, scholars have done a lot of research on the factors affecting air pollution [4–6], trying to explore ways to reduce air pollution and improve air quality. The impact of energy consumption on air pollution is particularly significant when electricity becomes an indispensable production factor for national economic development and the most important secondary energy source in modern socio-economic development.

Currently, China has taken the top position in both electricity production and consumption globally. In 2013, China’s installed power generation capacity surpassed that of the United States, reaching 5.39 trillion kilowatt-hours, ranking first in the world. Since 2003, China’s total electricity consumption has increased at an average annual growth rate of 8.71%. In 2011, China has become the country with the highest electricity consumption in the world, with a total of 4.7 trillion kilowatt-hours. Electricity consumption plays an important role in promoting the development of modernization and improving people’s living standards. China’s electricity consumption is primarily concentrated in the secondary industry, with the electricity consumption of the secondary industry accounting for 67.5% of the total electricity consumption in 2021 at 5613.1 billion kilowatt-hours. With the continuous increase in the total electricity consumption, China is facing severe challenges in environmental pollution, particularly in air pollution which has worsened [7].

In this context, how to scientifically and efficiently allocate electricity resources has become an important issue for China to promote comprehensive green transformation of economic and social development. This paper focuses on the relationship between power consumption and air pollution index, and thoroughly explores the internal mechanism of power consumption on air pollution in China, examining the impact of power consumption on different levels, in order to provide support for finding effective ways to prevent and control air pollution.

The contributions of this study are as follows: firstly, it enriches the research on air pollution by exploring the spatial correlation. Based on the construction of geographical spatial weight matrix, a spatial econometric model is employed to accurately identify the causal relationship between electricity consumption and air pollution. Secondly, a panel quantile regression is developed to more comprehensively describe the changes in electricity consumption’s impact on air pollution and its conditional distribution effects, eliminating estimation errors caused by outliers and revealing the marginal effects of electricity consumption on air pollution at different quantile points. Thirdly, the air pollution index used in this study is composed of four dimensions: sulfur dioxide, nitrogen oxides, particulate matter pollution, and carbon emissions. It provides an objective evaluation of regional air pollution conditions. Fourthly, the impact of electricity consumption on air pollution is studied from different temporal and geographical perspectives.

The remaining structure of this paper is as follows. Section 2 is literature review. Section 3 introduces the measurement of provincial air pollution index. Section 4 describes the research design to introduce the model, data and variables of this paper. Section 5 presents the basic empirical results and heterogeneity analysis, panel quantile regression and robustness test. Section 6 is the further analysis of spatial econometrics. Section 7 is the conclusion and countermeasures.

## 2. Literature review

The existing literature on air pollution research mainly includes the following aspects: Firstly, from the perspective of economic growth, Grossman and Krueger [8] found that when national income is low, the concentration of air pollutants such as sulfur dioxide tends to increase with per capita GDP growth. However, when national income is at a relatively high level, these pollutants tend to decrease with GDP growth. This phenomenon is similar to the relationship between income gap and per capita GDP, so it is called "Environmental Kuznets Curve" (EKC). Hartman and Kwon [9] and Panayotou [10] also verify this conclusion. Yin, Cheng [11], Yuanshu, Guodong [12] found that the environmental Kuznets curve also exists in China. Liu, Lei [13] tested whether there is an EKC curve between China’s economic growth and air pollution, and found that the environmental Kuznets curve of air pollution exists generally in cities across China. Some scholars also believe that the relationship between China’s economic growth and the environment does not conform to the EKC curve. Shao [14] found a positive "U-shaped" relationship between air pollution and economic growth based on PM2.5 data of Chinese provinces. Armeanu, Vintil [15] found that the relationship between economic growth and air pollution is nonlinear, but it does not conform to the general inverted "U" environmental Kuznets curve. Secondly, from the perspective of urbanization, York, Rosa [16] explored the relationship between urbanization and environmental pollution, and found that urbanization has a significant negative impact on the environment, primarily due to the rapid growth of population. Gallardo, Barraza [17] found that urbanization affects carbon emissions and PM10 concentrations, which in turn affects air pollution. Roberts [18] and Wang, Zhou [19] also reached the same conclusion. Thirdly, from the perspective of industrial structure and industrial agglomeration, Li, Lei [20] found that the change of industrial agglomeration and industrial structure has a significant impact on carbon dioxide emission reduction. Pei, Zhu [21] calculated the degree of urban environmental pollution based on the concentration of sulfur dioxide, and found that as industrial agglomeration increased, the level of urban pollution would also rise. Other scholars have examined the impact of sharing bicycles in cities [22], urban innovation capability [23], environmental regulation [24–27] on air pollution. In addition, research on air pollution measurement is also relatively rich. On the one hand, partial variables such as sulfur dioxide and PM2.5 concentration are used to measure urban environmental pollution level, such as Yu, Dai [28]. On the other hand, an air pollution index formulated by government agencies, such as the Ministry of Ecology and Environment in China, is adopted [29].

The research on electricity consumption mainly includes the following four aspects: firstly, the relationship between electricity consumption and economic growth. Liu, Guo [30] studied the impact of electricity consumption, capital and labor on economic growth from these three perspectives. They found that regional economic growth with restricted electricity consumption exhibits a demonstration effect, and electricity consumption significantly promotes economic growth. From the perspective of industrial structure, Zhang, Zhou [31] found that increasing electricity consumption would promote economic growth. When incorporating the influence of industrial structure, the extent of electricity consumption in promoting economic growth was significantly increased. Secondly, the relationship with technological innovation. Xie and Chen [32] proved that saving electricity through technological innovation will not only increase the total urban electricity consumption, but also increase the industrial and domestic electricity consumption. Thirdly, the relationship with urbanization rate. Xie, Wang [33] suggested that there is a long-term equilibrium between electricity consumption, urbanization level and economic growth, but only unidirectional Granger causality exists between urbanization level and electricity consumption. Fourthly, the relationship with foreign direct investment. Lei [34] believes that improving the level of electricity consumption is conducive to promoting China’s foreign direct investment level.

To sum up, through the review of existing research results, we found that there is some room for expansion in this field. Firstly, while there is abundant research on the factors affecting air pollution, empirical studies exploring the relationship between electricity consumption and air pollution from a spatial perspective are lacking. Secondly, it can be observed that both OLS regression used in dependent variable regression and panel data models tend to describe the average impact of electricity consumption on air pollution levels, overlooking the possibility of asymmetric distribution of air pollution conditions. This approach fails to accurately reflect the varying impacts of air pollution on production units at different air pollution levels and their differences. In reality, it is difficult for individual air pollution levels to satisfy the assumption of symmetric distribution. Thirdly, existing literature on air pollution research primarily uses partial relevant variables or air quality indices, lacking comprehensive indicators for an objective and systematic measurement. Fourthly, in terms of heterogeneity test, the effect of electricity consumption on air pollution from a heterogeneous perspective has not been thoroughly explored. This study analyzes the impact of electricity consumption on air pollution in China and provides solutions for optimizing air quality in China.

## 3. Measurement of provincial air pollution

### 3.1 Calculation method of provincial air pollution

This paper measures the air pollution index (AQI) with reference to the research results by Li, Hong [35] and Ma, Wang [36], taking 30 provinces (municipalities, districts) in China as the research sample. Four indicators are selected: sulfur dioxide, nitrogen oxide, particulate matter pollution, and carbon emissions. The air pollution index system is calculated using the method of entropy value from 2011 to 2020 (Table 1). The index can accurately reflect the level of air pollution. The relevant data of the selected indicators are from China Statistical Yearbook and China Environmental Statistics Yearbook.

**Table 1:** China’s air pollution measurement index system.

Total indicators	Level 1 indicators	Unit
Air pollution	Sulfur dioxide emissions	Tons
	Nitrogen oxide emissions	Tons
	Particulate emissions	Tons
	Carbon dioxide emissions	Ten thousand tons

The steps for the entropy method are as follows: firstly, the original sequence data needs to be standardized to eliminate interference from its magnitude, where in the standardization processing formula is as follows:

$$Z_{ij}^{\prime }=\frac{Z_{ij}-min(Z_{ij})}{Z_{ij}-max(Z_{ij})}\;(Positive\;indicator)$$

$$Z_{ij}^{\prime }=\frac{max(Z_{ij})-Z_{ij}}{max(Z_{ij})-min(Z_{ij})}\;(Negative\;Indicator)$$

i=1,2…n;j=1,2…m (1)

in Formula (1) *Z*_*ij*_ represented as the original sequence of the jth index in the ith year, $z_{ij}^{\prime }$ is the normalized value of the indicator. Min (*Z*_*ij*_) is that minimum of the original sequence, Max (*Z*_*ij*_) is that maximum of the original sequence. Secondly, the weight of each index is determined, and the steps are as follows:

step 1, calculate the relative proportion of the jth index in the ith year.

$$d_{ij}=\frac{Z_{ij}^{\prime }}{\sum_{i=1}^{m}Z_{ij}^{\prime }}$$ (2)

step 2, calculate that information entropy of J indexes.

$$e_{j}=-\frac{1}{lnm}\sum_{i=1}^{m}p_{ij}lnp_{ij}$$ (3)

step 3, calculate that weight of the jth index.

$$\omega_{j}=\frac{1-e_{j}}{\sum_{j=1}^{m}\left(1-e_{j}\right)}$$ (4)

step 4, calculate the composite index of the subsystem in the year i.


$$f(x)=\sum_{i=1}^{n}\omega_{j}\cdot d_{ij}$$ (5)

Among, ω_*j*_∈ [0,1]; $\sum_{j=1}^{n}\omega_{j}$ = 1, (1−*e*_*j*_) is the utility value of the jth index, the larger the value of (1−*e*_*j*_), the more important the indicator is.

### 3.2 Analysis of calculation results of air pollution at provincial level

#### 3.2.1 Overall level analysis

**Fig 1:**
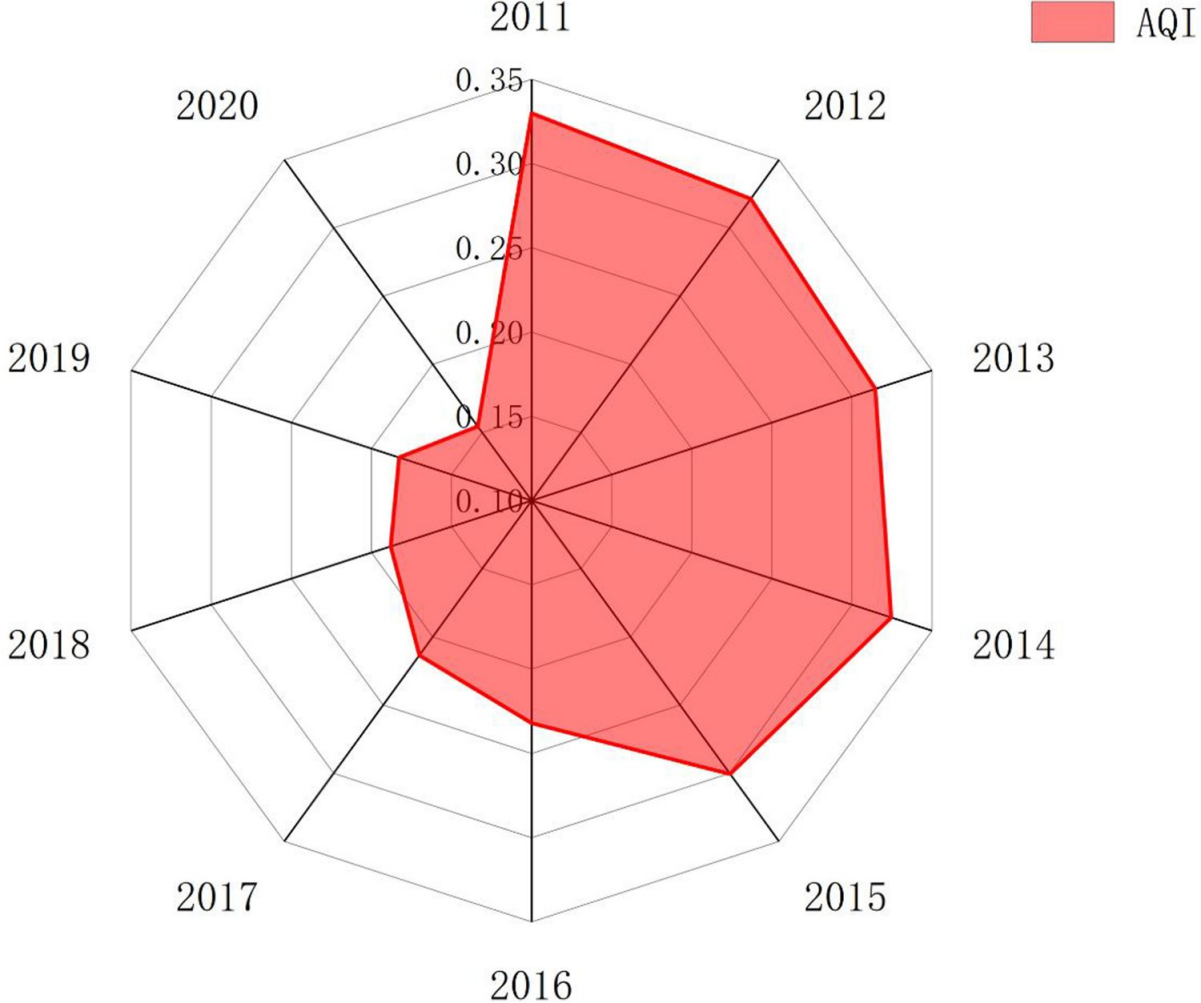
Temporal variation of air pollution.

The calculation results are shown in Fig 1. On the whole, the air pollution index shows a downward trend. Between 2011 and 2020, the air pollution index decreased by 53.15%, with an average annual decrease of 5.32%. From the perspective of changing trends, the air pollution index declined rapidly from 2015 to 2016, with an annual decline rate of 22.80%. It is possible that China’s first amendment of the Environmental Protection Law in 2014 further clarified the government’s regulatory responsibilities for environmental protection and delineated the red line for ecological protection. Previous environmental legislation prioritized the economy, but the new legislation revolves around building an ecological civilization and sustainable development concepts. From 2016 to 2020, air pollution index further decreased, possibly due to the China’s "Thirteenth Five-Year Plan" which emphasized green development as the primary task, implemented the strictest environmental protection system, and carried out in-depth actions to control atmospheric pollution, reducing air pollution and improving air quality. The concept of sustainable development was also incorporated into the planning process.

#### 3.2.2 Inter-provincial analysis

**Fig 2:**
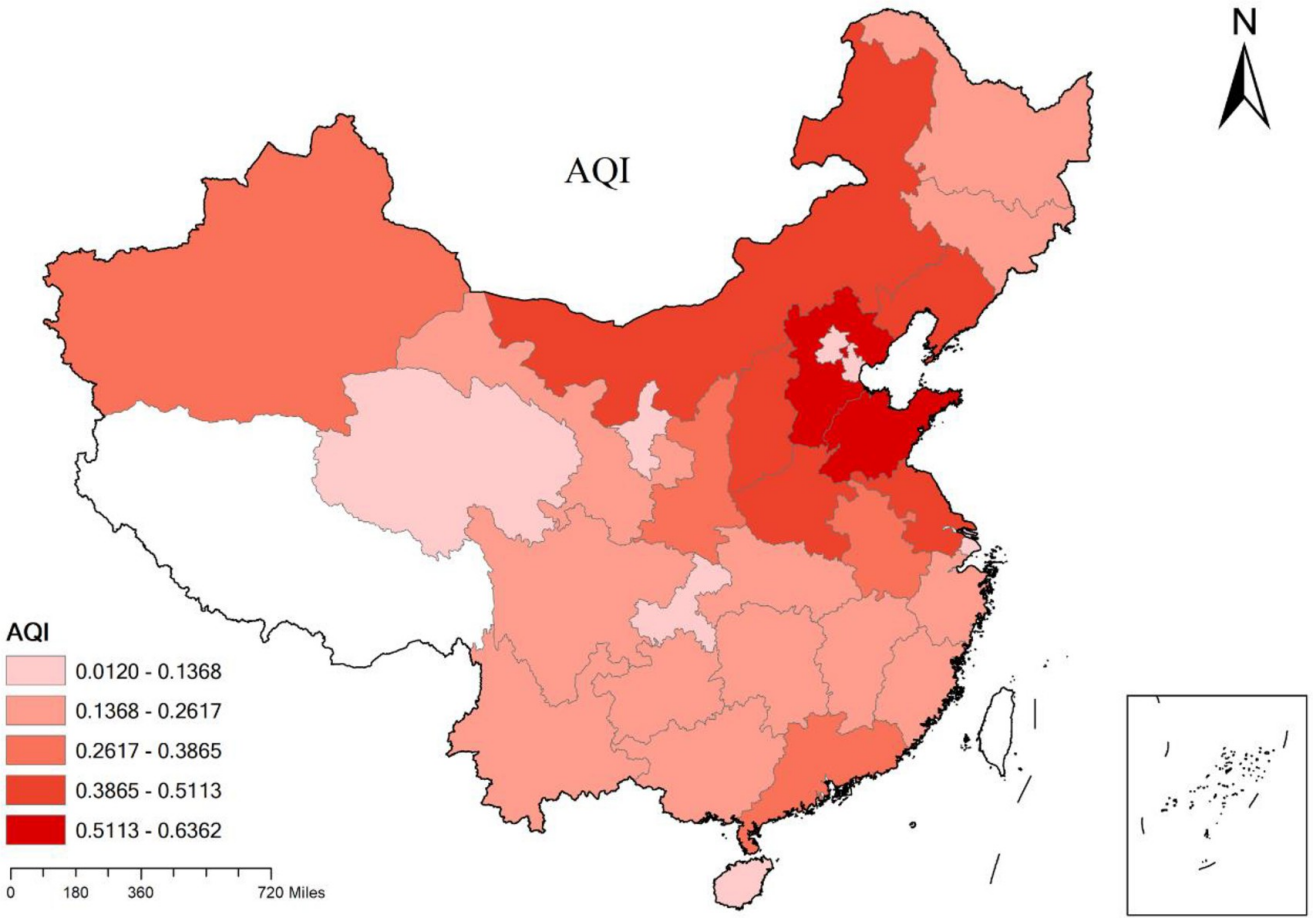
Average air pollution of each province and city. This figure is based on the standard map of the National Natural Resources Ministry’s Standard Map Service System. Http://bzdt.ch.mnr.gov.cn.

It can be seen from Fig 2 that there are significant differences in air pollution index between different provinces, with the air pollution index generally higher in the northern region than in the southern region. The region with the highest average air pollution index is Shandong Province, followed by Hebei Province and Inner Mongolia Autonomous Region. These three regions are mainly engaged in high energy-consuming and heavily polluting industries such as fuel processing and metal smelting. Their industrial structure is at a low end, which has a greater impact on air pollution. Furthermore, the thermal power generation accounts for up to 80% of the total electricity generation in Shandong Province. Currently, the primary source of electricity still comes from thermal power plants. This highly energy-intensive energy structure has caused severe pollution in Shandong. Hebei Province has a relatively low terrain and poor airflow, and the impact of factors such as coal-fired heating and traffic emissions in winter has led to the accumulation of a large amount of air pollutants. However, the climate in Inner Mongolia Autonomous Region is dry, with low annual precipitation but large evaporation. At the same time, the terrain is high and the wind is strong, which is prone to forming dust storms and causing serious air pollution. The two regions with the lowest air pollution levels are Beijing and Hainan. As the capital of China, Beijing has a relatively high-end industrial structure and stricter environmental protection standards, which to some extent control the sources of air pollution, resulting in relatively good air quality. Hainan Province takes the development model of regional ecological industry as the leading factor, with few industrial zones and high vegetation coverage. The oceanic airflow is also conducive to improving Hainan’s air quality.

## 4. Research design

### 4.1 Model building

To verify the impact of electricity consumption on air pollution in China’s provinces, a two-fixed effects benchmark regression model was constructed:

$$AQI_{it}=\alpha_{0}+\alpha_{1}LNEC_{it}+\alpha_{j}X_{it}+\mu_{i}+\mu_{t}+\varepsilon_{it}$$ (6)

where AQI_*it*_ represents the air pollution index of province i at time t, *LNEC*_*it*_ denotes the electricity consumption of province i at time t, X_*it*_ is the control variable that has an impact on air pollution for province i at time t, *u*_*i*_ represents the regional fixed effects, *u*_*t*_ represents the time fixed effects, and ε_*it*_ represents the error term.

Furthermore, this paper incorporates spatial factors into the model to study the spatial effects of electricity consumption on air pollution. Spatial econometric models include SAR, SEM, and SDM models.

$$SAR:{AQI}_{it}=\beta_{1}W{AQI}_{it-1}+\beta_{2}{LNEC}_{it}+\beta_{3}X_{it}+\mu_{i}+\mu_{t}+\varepsilon_{it}$$ (7)

$$SEM:{AQI}_{it}=\beta_{1}{LNEC}_{it}+\beta_{2}X_{it}+\lambda W+\mu_{i}+\mu_{t}+\varepsilon_{it}$$ (8)

$$SDM:{AQI}_{it}=\beta_{1}W{AQI}_{it-1}+\beta_{2}{LNEC}_{it}+\beta_{3}W{LNEC}_{it}+\beta_{4}X_{it}+\beta_{5}WX_{it}+\mu_{i}+\mu_{t}+\varepsilon_{it}$$ (9)

Where W represents the spatial weight matrix measuring the spatial weights between 30 provinces (cities, regions) in China, β_1_−β_5_ represent the coefficients to be estimated, and λ is the spatial error coefficient. Other coefficients are consistent with those in model (6).

With regard to the construction of spatial weight matrix W, there are two types of literatures. The first type is to construct the spatial weight matrix based on the inverse of geographical distance; the second type is to use economic distance matrix, assuming that the spatial correlation and economic development level are related, and using economic variables to calculate the weights. Since this study focuses on air pollution and electricity consumption, which are related to spatial distance, this paper chooses the geographical distance weight matrix.

In addition, in order to study the impact of electricity consumption on air pollution at different quantiles, this paper uses a panel quantile regression model. The panel quantile regression model can reveal the full picture of the conditional distribution of the dependent variable while not strictly assuming the residual term and making the estimation results less susceptible to extreme values, making the estimation results more robust. Based on the research findings of Machado and Santos Silva [37], the fixed effect panel quantile model is established, and the Formula (6) can be transformed into:

$$Q_{{AQI}_{it}}(t|{LNEC}_{it},X_{it})=b(t){LNEC}_{it-1}+g(t)X_{it}+\mu_{i}+\mu_{t}+\varepsilon_{it}$$ (10)

### 4.2 Variable description

#### 4.2.1 Explained variable

Air pollution index (AQI) is the explanatory variable in this paper, which can quantitatively describe the air pollution of each province. The focus of AQI is on the impact of air pollution on health after living in the air environment for a period of time. The larger the AQI value, the more severe the environmental pollution is.

#### 4.2.2 Explain variables

**Fig 3:**
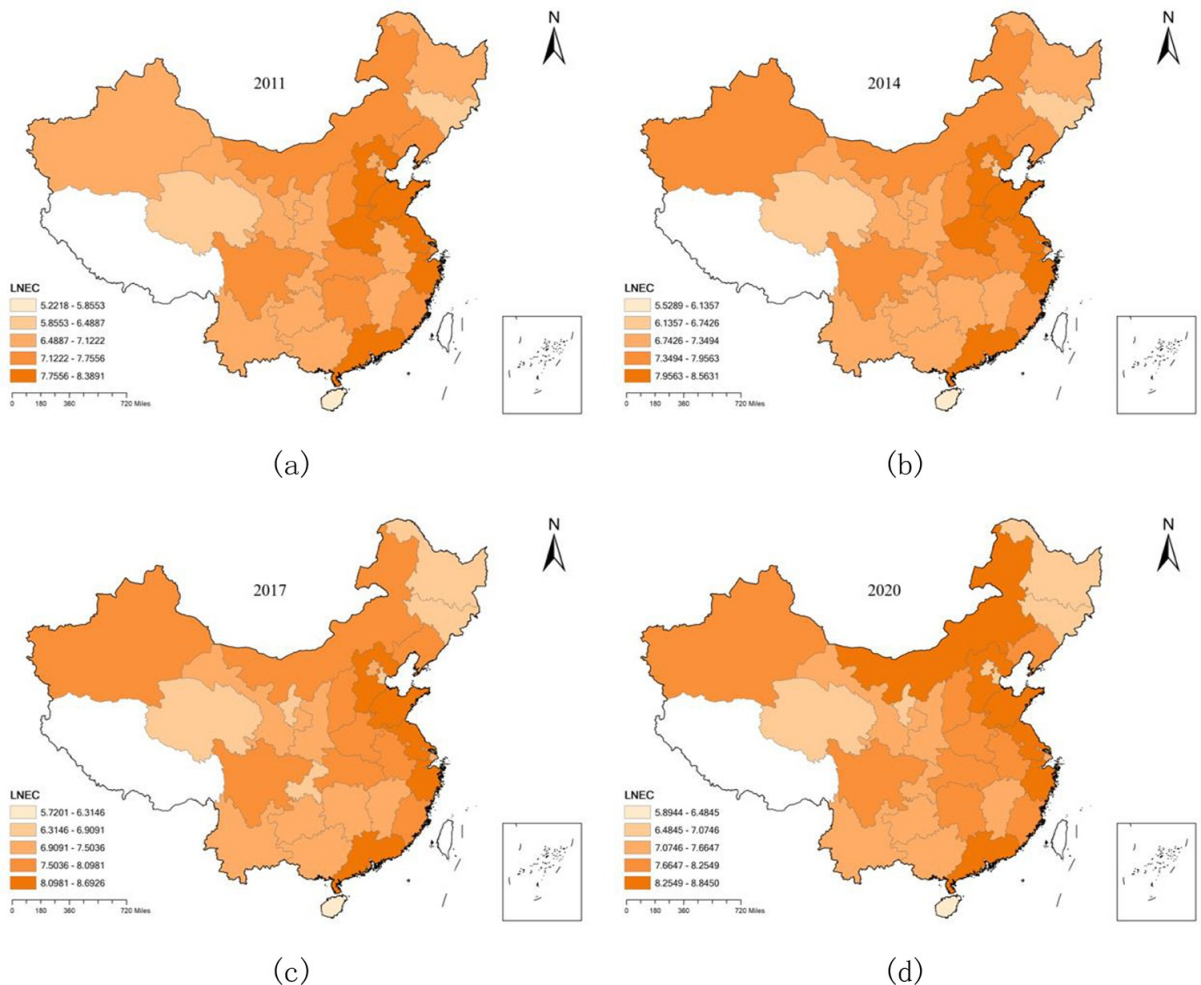
China’s electricity consumption in 2011 (a), 2014 (b), 2017 (c) and 2020 (d). This figure is based on the standard map of the National Natural Resources Ministry’s Standard Map Service System. Http://bzdt.ch.mnr.gov.cn.

The core explanatory variable in this paper is electricity consumption, which is measured by the logarithm of the annual electricity consumption of each province (city, district). The electricity consumption of 30 provinces (cities, districts) in some years is shown in Fig 3.

#### 4.2.3 Control variable

Air pollution can also be influenced by other factors. Referring to the research results of Zheng, Peng [38] and Wu, Deng [39], the following five control variables were selected: (1) foreign investment level (FDI), using the proportion of actual foreign investment in provincial GDP as an proxy for FDI level; (2) industrial structure level (RIS), measured by the proportion of the tertiary industry output value to GDP, which consumes less energy and produces less pollution than the first and second industries; the higher the proportion, the higher the level of industrial structure; (3) government intervention level (GOV), measured by the proportion of government financial expenditure to annual GDP; (4) living environment level (LIVING), measured by the per capita green space area, which is an important indicator reflecting urban residents’ living environment and pollution; usually speaking, an increase in the per capita green space area will reduce pollutants in production; and (5) human capital level (HC), measured by the proportion of college students with a degree or above in each province’s total population.

#### 4.2.4 Data sources and descriptive statistics

This paper selects the panel data of 30 provinces (cities, districts) in China from 2011 to 2020.The data in this paper comes from China Statistical Yearbook, China Energy Statistical Yearbook, China Environmental Statistical Yearbook and provincial statistical yearbooks. In addition, some variables were standardized in this paper, and the data analysis was performed using Stata 16.0 software. The descriptive statistics of the variables involved are shown in Table 2.

**Table 2:** Descriptive statistics of variables.

Variables	Symbol	Number of samples	Mean value	Standard deviation	Minimum value	Maximum value
Air pollution	AQI	300	0.2561	0.1776	0.0056	0.8107
Electricity consumption	LNEC	300	7.3790	0.6847	5.2219	8.8451
Level of foreign investment	FDI	300	0.0194	0.0183	0.0001	0.1210
Level of industrial structure	RIS	300	0.4916	0.0897	0.3266	0.8373
Degree of government intervention	GOV	300	0.2644	0.1145	0.1196	0.7583
Level of living environment	LIVING	300	2.5500	0.2127	1.9473	3.0469
Level of human capital	HC	300	0.0201	0.0053	0.0080	0.0413

## 5. Empirical analysis

### 5.1 Correlation analysis

The correlation analysis results of the variables are shown in S1 File. It can be seen that there is a significant correlation between air pollution and electricity consumption. From the statistical point of view, the critical value of the correlation coefficient is 0.7, and if it exceeds 0.7, it is considered that there is a linear relationship between the variables. According to the table, most of the correlation coefficients between variables are less than 0.7, indicating that there is no serious multicollinearity problem between variables.

### 5.2 Model estimation results

The OLS and fixed effect models are used to regress electricity consumption and air pollution. The regression results of OLS and fixed effect models without time and regional fixed effects are listed in (1) and (2) of Table 3. The results show that the coefficients of electricity consumption are 0.1341 and 0.0564 respectively, which are significant at the 1% level. In order to avoid the impact of time and geographical factors on the model, column (3) was added with time and geographical fixed effects. The estimated coefficient of electricity consumption was also significantly positive at the 1% level, indicating a significant positive correlation between electricity consumption and air pollution changes. This suggests that for every increase in electricity consumption by 1%, the air quality index will increase by 0.0649%. Therefore, electricity consumption can significantly increase the level of air pollution. The reason is that China’s long-term implementation of the low electricity price policy has reduced the production cost of high energy consumption industries, which will expand the production scale of high energy consumption industries [40]. At the same time, according to the "rebound effect", as electricity consumption levels increase, the electricity industry will adopt new technologies, resulting in a decrease in electricity consumption costs and an increase in total electricity consumption. This leads to an increase in pollutant emissions, ultimately leading to an increase in air quality index. Secondly, electricity is a secondary energy source, and consuming it can have negative impacts on the environment. However, by undergoing processing to become an alternative for other high-polluting energy sources, electricity reduces its negative environmental impact to a certain extent [41]. The rapid growth of electricity consumption has resulted in the "rebound effect" having a greater impact on the environment than the "substitution effect."

**Table 3:** Regression results of electricity consumption on air pollution.

	(1)	(2)	(3)
	AQI	AQI	AQI
LNEC	0.1341***	0.0564**	0.0649*
	(9.5509)	(2.5209)	(1.6512)
FDI	1.0640**	-0.0978	-0.4902
	(2.5103)	(-0.3187)	(-1.5687)
RIS	-0.8582***	-1.1294***	-0.6805***
	(-10.1918)	(-9.1226)	(-3.3008)
GOV	-0.0692	-0.1844	-0.1425
	(-0.8044)	(-1.4270)	(-0.8179)
LIVING	0.0018	-0.0692*	-0.0407
	(0.0523)	(-1.7558)	(-0.9791)
HC	-2.8315*	-9.3169***	-6.4205**
	(-1.6596)	(-4.4225)	(-2.2950)
_cons	-0.2613*	0.8094***	0.4188
	(-1.8366)	(5.4897)	(1.3788)
City	NO	NO	YES
Year	NO	NO	YES
*R* ^2^	0.5729	0.6063	0.6833
N	300.0000	300.0000	300.0000

*z* statistics in parentheses,* *p* < 0.1

** *p* < 0.05

*** *p* < 0.01

### 5.3 Heterogeneity analysis

#### 5.3.1 Time difference

As the sample survey period covers the years from 2011 to 2020, it experiences two phases: “the Eleventh Five-Year Plan” and "the Thirteenth Five-Year Plan". During the thirteenth five-year plan period, there is more emphasis on ecological protection compared to the twelfth five-year plan. This includes significantly increasing the task requirements for ecological protection targets, while also strengthening the linkage and synergy between pollution prevention and control and ecological protection, focusing on air pollution prevention and improvement. Therefore, this paper divides the sample into two time periods: 2011–2015 and 2016–2020 to compare the impact of electricity consumption on air pollution in different periods and study the effect of policy changes on green total factor productivity.

Table 4 displays the regression results for the two time periods. The coefficients of electricity consumption on air pollution index productivity are positive in both time periods, but the impact of electricity consumption on air pollution is more significant in the years from 2011 to 2015. This may be due to the implementation of stricter environmental regulations and the establishment of public monitoring and early warning mechanisms for environmental pollution by local governments during the "Thirteenth Five-Year Plan". As a result, the cost of pollution has increased for enterprises, who must consider reducing their electricity consumption through management system and technological innovation. Meanwhile, high energy consumption and high pollution enterprises have exited the market due to strict environmental regulations, and those that remain will invest their electricity resources in industries with high technology and environmental benefits. This reduces air pollution.

**Table 4:** Regression results of electricity consumption on air pollution at different times.

	2011–2015	2016–2020
LNEC	0.0898***	0.0269
	(5.5244)	(0.4110)
FDI	-0.1613	-0.8078
	(-0.8482)	(-1.5872)
RIS	0.1038	-0.7010***
	(1.2245)	(-2.6957)
GOV	0.1444	-0.4845**
	(1.3180)	(-2.4945)
LIVING	-0.0126	0.0161
	(-0.6687)	(0.3271)
HC	1.5916	1.5633
	(0.8566)	(0.4514)
_cons	-0.3858***	0.4660
	(-2.9624)	(0.8771)
City	YES	YES
Year	YES	YES
*R* ^2^	0.5749	0.6242
N	150.0000	150.0000

*z* statistics in parentheses,* *p* < 0.1

** *p* < 0.05

*** *p* < 0.01

#### 5.3.2 Geographical differences

Since population has been an important factor influencing environmental pollution, the "Hu Line” divides China into two relatively stable regions with different population densities. The southeast region accounts for 43% of China’s land area but contains over 90% of its population, and has a higher level of economic development, while the northwest region is sparsely populated and economically underdeveloped. Therefore, this section divides the 30 provinces and municipalities into the southeast and northwest regions based on the "Hu Line", as shown in Table 5. It is evident that electricity consumption in the southeast region has a more significant impact on increasing air pollution levels than in the northwest region. This means that the southeast region bears greater responsibility for the impact of electricity consumption on air pollution. Furthermore, it indicates that differences in population density and economic and social development can lead to different effects of electricity consumption on air pollution. In order to improve air quality, provinces and municipalities in the southeast region need to optimize their energy consumption structure and reduce pollutant emissions.

**Table 5:** Regression results of electricity consumption on air pollution in different geographical locations.

	Northwest side	Southeast side
LNEC	-0.0264	0.2732***
	(-0.6427)	(3.5212)
FDI	1.8290*	-0.8900**
	(1.8743)	(-2.2028)
RIS	-0.7947***	-0.9643***
	(-2.7851)	(-3.4530)
GOV	-0.0296	-0.2127
	(-0.1419)	(-0.7163)
LIVING	0.0801	-0.0558
	(1.1255)	(-1.0499)
HC	19.1182***	-13.6230***
	(2.9687)	(-4.2263)
_cons	0.3351	-0.7520
	(1.0199)	(-1.2201)
City	YES	YES
Year	YES	YES
*R* ^2^	0.7907	0.7058
N	100.0000	200.0000

*z* statistics in parentheses,* *p* < 0.1

** *p* < 0.05

*** *p* < 0.01

### 5.4 Panel quantile regression

The panel quantile model can estimate the corresponding regression equation for each quantile point, and the coefficient estimates can reveal the marginal impact of different levels of electricity consumption on air pollution index. Based on Xu and Lin [42] research results, five quantile points (0.1, 0.25, 0.5, 0.75, 0.9) were set to estimate the distribution of electricity consumption on air pollution index under different conditions. The estimation results are shown in Table 6. The regression results show that the coefficients of electricity consumption at all five quantile points are positive, and the significance test at the 1% level indicates that electricity consumption has a promoting effect on air pollution index. Higher air pollution index reflects the severity of air pollution. Specifically, as the quantile point increases from 0.1 to 0.9, the coefficient of electricity consumption on air pollution index increases from 0.0618 to 0.1976. This means that with the increase in quantile point, the quantile regression coefficient also continues to increase, and the positive impact of electricity consumption on air pollution is getting larger. This is because higher levels of electricity consumption have a greater negative impact on the environment. Therefore, it is necessary to increase the proportion of clean energy such as solar energy and gradually reduce the proportion of fossil energy such as coal-fired power generation.

**Table 6:** Panel quantile model regression estimation Results of electricity consumption on air pollution.

	0.1	0.25	0.5	0.75	0.9
LNEC	0.0604***	0.0832***	0.0875***	0.1199***	0.2398***
	(12.8742)	(65.4236)	(8.8066)	(29.0280)	(16.9754)
FDI	-0.0633	0.2959***	-0.2629***	0.1833**	-0.4106
	(-0.7359)	(6.8630)	(-2.6072)	(2.0876)	(-1.3204)
RIS	-0.4997***	-0.5879***	-0.5790***	-0.7707***	-0.8374***
	(-11.0773)	(-107.5515)	(-19.4712)	(-32.3187)	(-42.0759)
GOV	-0.1525***	-0.2169***	-0.2689***	-0.4503***	-0.4079***
	(-12.3982)	(-14.8352)	(-8.3724)	(-32.6874)	(-6.8037)
LIVING	-0.0581***	-0.0320***	-0.0348	-0.0731***	0.0303
	(-6.8279)	(-3.3971)	(-0.7404)	(-10.0153)	(1.5460)
HC	-1.6250**	-1.7194***	-1.4410	-5.0432***	-4.8190***
	(-2.4831)	(-2.8340)	(-1.5173)	(-23.3440)	(-3.7535)
City	YES	YES	YES	YES	YES
Year	YES	YES	YES	YES	YES
N	300.0000	300.0000	300.0000	300.0000	300.0000

*z* statistics in parentheses,* *p* < 0.1

** *p* < 0.05

*** *p* < 0.01

### 5.5 Robustness test

In order to ensure the reliability of this study, t robustness tests were conducted for the baseline regression model in four aspects: First, we replaced the core explanatory variable with the logarithmic measure of PM_2.5_ concentration in each province.PM_2.5_ data are from the Atmospheric Composition Analysis Group of Dalhousie University, as shown in Column (1) of Table 7. Second, after excluding the provinces of Qinghai, Hainan, and Jilin with relatively low levels of electricity consumption, we re-ran the regression model and the estimation results are shown in Column (2) of Table 7. Third, after excluding the four municipalities of Beijing, Tianjin, Shanghai, and Chongqing from the sample and re-running the regression model, the results are shown in Column (3) of Table 7. Finally, in order to mitigate potential confounding effects and obtain unbiased consistent estimates, this paper adopts the Generalized Linear Mixed Model (GMM) proposed by Arellano and Bond [43]. When using the GMM model for estimation, we test the validity of the instrumental variables used in the model and conduct autocorrelation tests on its residual series. The AR (2) test results show that all models pass the autocorrelation test, and there is no second-order sequence autocorrelation, thus verifying the validity of the estimation. The Sargan test results are all greater than 0.1, indicating that the instrumental variable is not subject to overidentification. Therefore, it can be concluded that the GMM estimation method used in this study is reasonable. The estimation results are shown in Column (4) of Table 7.

**Table 7:** Robustness test of electricity consumption to air pollution.

	(1)	(2)	(3)	(4)
L.AQI				0.7281***
				(19.2164)
LNEC	0.1316**	0.0775*	0.1053**	0.0587***
	(2.0563)	(1.7860)	(2.4648)	(2.7571)
FDI	-0.6979	-0.6349*	-0.2202	0.1519
	(-1.3729)	(-1.9207)	(-0.5336)	(0.8907)
RIS	0.1548	-0.5005**	-0.6115***	-0.7946***
	(0.4616)	(-2.1801)	(-2.6451)	(-7.5006)
GOV	0.0521	-0.0995	-0.2210	-0.2161
	(0.1837)	(-0.4605)	(-1.1258)	(-1.4381)
LIVING	0.0988	0.0177	-0.0805	0.0326
	(1.4596)	(0.3873)	(-1.5520)	(1.0147)
HC	-2.1328	-6.9629**	-0.7717	-0.9546
	(-0.4686)	(-2.3230)	(-0.2176)	(-0.4849)
_cons	2.5597***	0.1259	0.1201	0.0137
	(5.1804)	(0.3656)	(0.3592)	(0.0786)
AR (2)				0.2151
Sargan				0.0196
City	YES	YES	YES	YES
Year	YES	YES	YES	YES
*R* ^2^	0.8251	0.7020	0.6985	
N	300.0000	270.0000	260.0000	240.0000

*z* statistics in parentheses, * *p* < 0.1

** *p* < 0.05

*** *p* < 0.01

The robustness test results in Table 7 indicate that the significance and signs of the regression model are consistent with those of the benchmark regression, except for the varying degree of electricity consumption coefficients. Therefore, the estimation results of this paper’s regression model are robust.

## 6. Further discussion

### 6.1 Research on spatial effect

#### 6.1.1 Spatial autocorrelation test

Based on the above elaboration of the relationship between electricity consumption level and air pollution, this paper further explores whether there is a spatial connection and influence between changes in electricity consumption and air pollution. Firstly, we need to understand the spatial correlation and degree of correlation between the two factors. We can test this by calculating the Moran’s index. The calculation formula for Moran’s index is:

$$I=\frac{\sum_{i=1}^{n}\sum_{j=1}^{n}W_{ij}(Y_{i}-\bar{Y})(Y_{j}-\bar{Y})}{S^{2}\sum_{i=1}^{n}\sum_{j=1}^{n}W_{ij}}$$ (11)

where $S^{2}=\frac{1}{n}\sum_{i=1}^{n}{\left(Y_{i}-\bar{Y}\right)}^{2}$, $\bar{Y}=\frac{1}{n}\sum_{i=1}^{n}Y_{i}$, I indicates the Moran’s index, n is the number of provinces in China (30), *W*_*ij*_ is the spatial weight matrix, and *Y*_*i*_ represents the air pollution index of a region. $\bar{Y}$ is the average value of the regional air pollution index. The calculated results are shown in Table 8.

**Table 8:** Global Moran index of air pollution in Chinese provinces.

Year	I value	Z value	P value	Year	I value	Z value	P value
2011	0.196	2.422	0.015	2016	0.170	2.151	0.031
2012	0.194	2.397	0.017	2017	0.161	2.044	0.041
2013	0.194	2.397	0.017	2018	0.119	1.603	0.100
2014	0.201	2.479	0.013	2019	0.117	1.586	0.113
2015	0.209	2.571	0.010	2020	0.132	1.743	0.081

From the results in Table 8, it can be observed that most provinces pass the significance test and exhibit positive correlation, indicating that there is significant spatial correlation between air pollution in Chinese provinces. Therefore, when studying the impact of electricity consumption on air pollution, the spatial factor should be taken into account.

**Fig 4:**
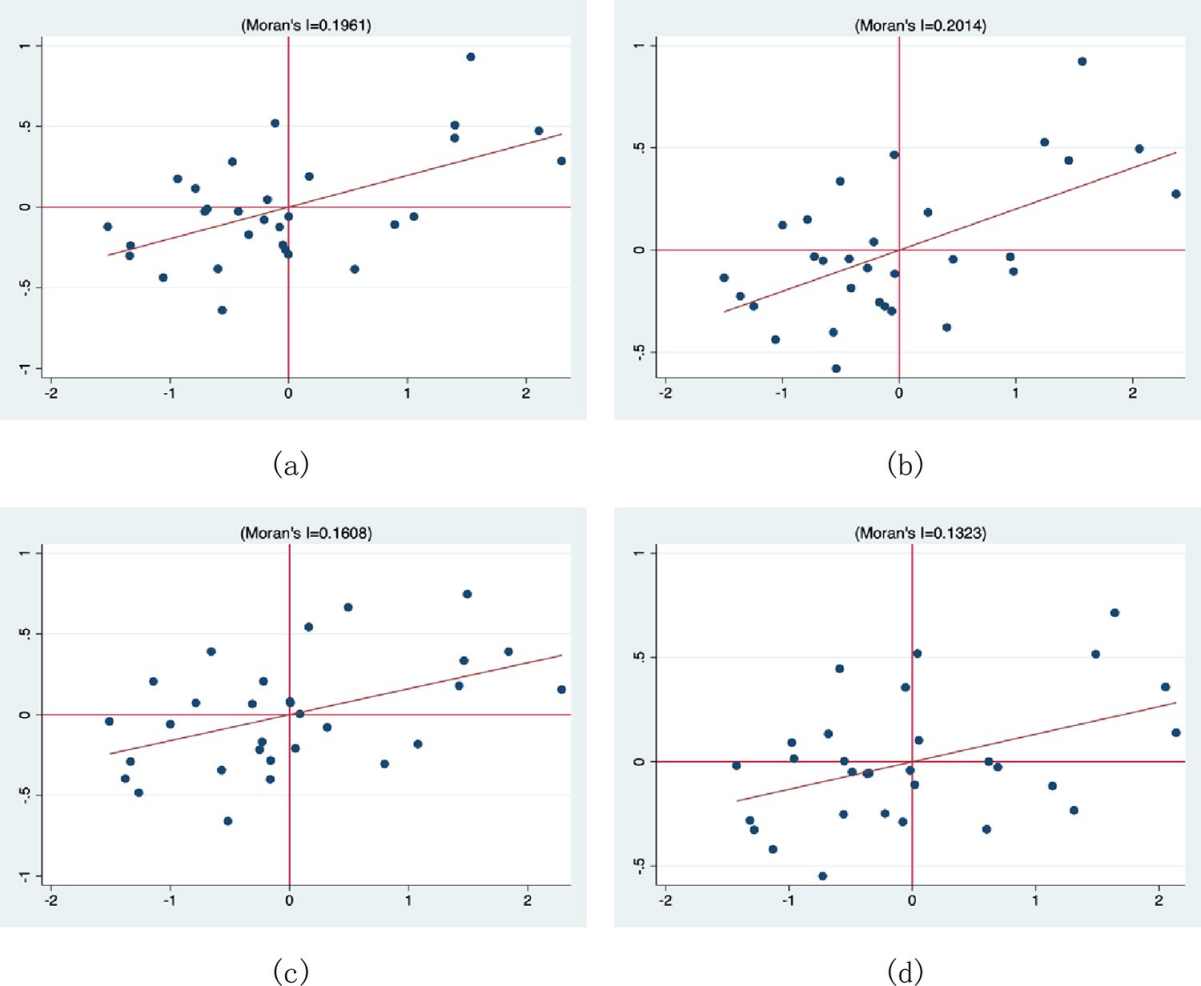
Scatter plot of Moran index in 2011 (a), 2014 (b), 2017 (c) and 2020 (d).

To better reflect the spatial aggregation of air pollution index, this paper takes 2011, 2014, 2017 and 2020 as examples, and draws Moran’s Index scatter plots, as shown in Fig 4. The horizontal axis represents the deviation of air quality index from the mean, and the vertical axis represents the spatial lag term of air quality index. Each scatter plot has four quadrants, representing four spatial aggregation properties.

As can be seen from Fig 4, there are spatial aggregation effects of air pollution index levels in different regions, mainly showing two types of aggregation characteristics: "low-low" and "high-high", which indicate that areas with high air pollution index levels tend to cluster together, and areas with low air pollution index levels also tend to cluster together. Therefore, the model built in this paper is reasonable.

#### 6.1.2 Spatial spillover effects

Before conducting spatial econometric analysis, it is necessary to conduct correlation tests to determine the optimal spatial econometric model. The test results are shown in Table 9. According to the research result of Yandell [44], the first step was to conduct an LM test. The results showed that both the SEM and SAR models had significant LM values at the 1% level, and the Robust LM value was also significant at the same level. This rejection of the null hypothesis of spatial error and spatial lag effect indicates that both the SEM and SAR models are suitable. In addition, the Hausman test showed that fixed effects were preferred over random effects. Further LR tests and Wald statistics results passed the 1% significance test, indicating that the SDM model was effective.

**Table 9:** Joint significance test results of LM, LR and Wald.

Significance test	Statistical value	P value
LM-SEM	122.413	0.0000
Robust LM-SEM	21.538	0.0000
LM-SAR	105.179	0.0000
Robust LM-SAR	4.303	0.0380
LR-SEM	30.51	0.0000
LR-SAR	23.61	0.0006
Wald-SEM	32.01	0.0000
Wald-SAR	24.20	0.0005

This paper analyzes the regression results of SAR model, SEM model and SDM model based on the geographical distance weight matrix. The results are shown in Table 10. From the results, both the spatial regression coefficients and spatial error coefficients were significant at the 1% level, indicating that changes in air pollution in one region will affect changes in neighboring regions. The regression coefficient for the explanatory variable electricity consumption was positive and passed the 10% significance test in all three models. Specifically, the regression coefficient for electricity consumption in the SDM model was 0.0522, which means that an increase in electricity consumption by 1% would result in a 0.0522% increase in air pollution index. This shows that an increase in electricity consumption leads to an increase in air pollution, which is detrimental to improving air pollution levels. The WxLNEC coefficient was significant positive, indicating that increases in local electricity consumption through spatial spillover effects lead to an increase in nearby regions’ air pollution index. From the autoregressive coefficients, it can be seen that the value of ρ was significant at the 1% level and positive, indicating that there is a significant spatial autocorrelation in the air pollution index. There is a positive correlation between air pollution in this region and neighboring regions.

**Table 10:** Regression results of spatial econometric model.

	SAR		SEM		SDM	
LNEC	0.1028***	0.0595*	0.0898***	0.0507*	0.1078***	0.0522*
	(5.4161)	(1.8571)	(3.6206)	(1.5533)	(5.2647)	(1.6529)
FDI	-0.2452	-0.2568	-0.1888	-0.2202	-0.1584	0.0688
	(-1.0282)	(-1.0030)	(-0.8276)	(-0.8609)	(-0.6292)	(0.2402)
RIS	-0.5359***	-0.5137***	-0.4353***	-0.3429**	-0.4432***	-0.5722***
	(-4.9599)	(-3.0374)	(-3.1047)	(-2.0179)	(-3.3483)	(-3.1533)
GOV	-0.1181	-0.1862	-0.1773	-0.1558	-0.1934*	-0.2468*
	(-1.1364)	(-1.3099)	(-1.5964)	(-1.0971)	(-1.6993)	(-1.6911)
LIVING	-0.0296	-0.0498	-0.0453	-0.0454	-0.0170	-0.0565
	(-0.9629)	(-1.4669)	(-1.5255)	(-1.4705)	(-0.5436)	(-1.5631)
HC	-4.9295***	-6.3395***	-6.6805***	-6.6159***	-6.5063***	-8.2944***
	(-2.9019)	(-2.7805)	(-3.1427)	(-2.8431)	(-3.1604)	(-3.6197)
W*LNEC					-0.0913*	0.2517**
					(-1.8680)	(2.0575)
W*FDI					-0.0051	-1.4776**
					(-0.0094)	(-2.2036)
W*RIS					-0.2620	-2.0172***
					(-1.1493)	(-4.1318)
W*GOV					0.0510	0.3464
					(0.2293)	(1.0453)
W*LIVING					0.1515**	0.0847
					(1.9810)	(0.8844)
W*HC					5.8448*	-1.8798
					(1.8122)	(-0.3568)
*ρ*/*λ*	0.7296***	0.6481***	0.8400***	0.6307***	0.7215***	0.6151***
	(13.8653)	(8.6664)	(20.5181)	(7.9649)	(12.2628)	(7.7526)
*σ* ^2^	0.0018***	0.0016***	0.0018***	0.0017***	0.0018***	0.0015***
	(11.2564)	(11.7054)	(11.0227)	(11.6991)	(11.1153)	(11.7391)
LogL	446.4722	524.4189	446.4722	520.9714	451.8677	536.2244
City	NO	YES	NO	YES	NO	YES
Year	NO	YES	NO	YES	NO	YES
*R* ^2^	0.3557	0.3300	0.5160	0.4737	0.4891	0.1291
N	300.0000	300.0000	300.0000	300.0000	300.0000	300.0000

*z* statistics in parentheses, * *p* < 0.1

** *p* < 0.05

*** *p* < 0.01

From the perspective of control variables, the estimated results of the main variables are basically in line with theoretical expectations. The SDM model results in Table 10 indicate that the level of industrial structure, government intervention and human capital are significantly negatively correlated with the local air pollution index.

It is possible that an increase in the industrial structure level indicates that the proportion of the tertiary industry increases, which can inhibit the development of high-pollution and high-energy consumption industries and thus improve air quality. An improvement in government intervention level demonstrates that the government pays more attention to environmental protection and can set up environmental laws, regulations, and other standards to increase environmental standards. Economic entities such as enterprises have no choice but to comply with the regulations such as emission standards and technological standards set by the government, otherwise they will be severely punished. An increase in human capital level can enhance citizens’ awareness of environmental protection, and the public will consciously exercise their rights to environmental supervision and litigation enshrined by environmental law, giving pressure to the government and environmental offenders to remove environmental hazards and contribute to reducing air pollution. The lack of correlation between foreign investment level and living environment level and the local air pollution index may be due to the fact that both indicators have reached a stable level in various provinces of China, with small annual growth rates, resulting in a limited impact on the air pollution index. From the interaction coefficient of control variables, it can be seen that an increase in electricity consumption will also lead to an increase in the air pollution level in neighboring regions. However, an improvement in foreign investment level, industrial structure level, and government intervention level will improve the air pollution level in neighboring regions.

Due to the presence of endogenous spatial interaction effects in spatial econometric models, this paper refers to the research of Lesage and Pace [45] to decompose the effects. The spillover effects of SDM models are shown in Table 11. From the direct effect perspective, the coefficient of electricity consumption is significantly positive at the 5% level, indicating that an increase in electricity consumption will lead to an increase in air pollution index. From the indirect effect perspective, the coefficient of electricity consumption is positive and passes a 5% significance test, indicating that an increase in the electricity consumption level in neighboring regions can also lead to an increase in the local air pollution index. This also indicates that there is a spatial spillover effect of air pollution.

**Table 11:** Decomposition results of spillover effect of spatial Durbin model.

	Direct effect	Indirect effect	Total effect
LNEC	0.0977**	0.7192**	0.8169**
	(2.3400)	(2.0494)	(2.1553)
FDI	-0.1760	-3.7604**	-3.9364*
	(-0.5399)	(-1.9987)	(-1.8898)
RIS	-0.9158***	-5.8620***	-6.7778***
	(-3.9579)	(-3.1209)	(-3.3158)
GOV	-0.2230	0.4706	0.2475
	(-1.4128)	(0.5366)	(0.2552)
LIVING	-0.0471	0.1315	0.0844
	(-0.9844)	(0.4493)	(0.2539)
HC	-9.2232***	-16.7485	-25.9717
	(-3.6111)	(-1.1363)	(-1.6096)

*z* statistics in parentheses, * *p* < 0.1

** *p* < 0.05

*** *p* < 0.01

## 7. Conclusion and policy implications

This paper explores the impact mechanism of electricity consumption on provincial air pollution through panel data from 30 provinces (cities, districts) in 2011–2020. The results show that electricity consumption has a significant positive effect on air pollution index, with an increase of 0.0649% for every 1% increase in electricity consumption. From different time periods, the promotion effect of electricity consumption on air pollution will decrease as environmental protection efforts are enhanced. From different geographical locations, the southeast side of the "Hu Line" has a greater promoting effect on air pollution than the northwest side. Additionally, the panel quantile model results show that as electricity consumption increases, it will also increase its impact on provincial air pollution. Further analysis reveals that there is spatial spillover effect of electricity consumption on air pollution, indicating that electricity consumption not only causes air pollution in the region but also affects neighboring regions. From the perspective of spatial effects decomposition, the local air pollution will also be influenced by electricity consumption in neighboring regions.

Based on these findings, this paper proposes the following countermeasures and suggestions. Firstly, it is appropriate to raise electricity prices and impose punishing electricity tariffs on high-energy consumption and high-pollution industries in order to increase production costs. China has long pursued a policy of low electricity prices to support economic development, which has led to the expansion of high-energy consumption industries and overcapacity. An increase in electricity prices will reduce the competitiveness of thermal power generation to some extent, while enhancing the competitiveness of clean energy such as wind power, photovoltaic power, nuclear power, and hydropower. Promoting green transformation of the electricity structure can help optimize air pollution. Secondly, clean energy generation promotes the upgrading of electricity consumption structure, which is an important way to affect air pollution emissions. However, China is still a major consumer of fossil energy mainly composed of coal. One important reason for this is that the industrial structure mainly composed of the secondary industry will lead to more energy consumption from fossil fuels. Therefore, it is necessary to increase the proportion of the tertiary industry in order to weaken the adverse impact of the secondary industry on air pollution. We should vigorously develop strategic emerging industries such as new energy and clean energy, and cultivate new growth points with low energy consumption, low emissions, and high quality. Thirdly, raising the education level of citizens can enhance their environmental awareness. When the environmental pollution caused by electricity consumption threatens citizen health, citizens will consciously exercise their rights to supervise and litigate under environmental protection laws, exert pressure on governments and violators of environmental protection laws, and monitor them to eliminate environmental hazards. Fourthly, air pollution has a significant spatial agglomeration characteristic, therefore, the control of air pollution requires strengthening communication and cooperation between provinces, establishing an integrated pollution prevention and control mechanism, as well as an information sharing platform to work together to improve air quality.

## Supporting information

S1 File Appendix of the correlation analysis. It shows that there is no serious multicollinearity problem between variables, which are also mentioned in the main text.
(DOCX)

S2 File Accessible channels of all original data used in this paper are showing in this file. (DOCX)

S3 File Original data used in the calculations of all figures and tables. It contains the original data of AQI, LNEC etc.
(XLSX)
