# Supplementary material for: Evaluation the impact of electricity consumption on China’s air pollution at the provincial level
Source: PLoS One. 2024 Apr 16;19(4):e0301537. doi: 10.1371/journal.pone.0301537 (PMC11020704; doi:10.1371/journal.pone.0301537)
Supplement: S1 File — It shows that there is no serious multicollinearity problem between variables, which are also mentioned in the main text. (DOCX) [file pone.0301537.s001.docx]

Table 3. Correlation analysis

|  | AQI | LNELE | FDI | RIS | GOV | LIVING | HC |
| --- | --- | --- | --- | --- | --- | --- | --- |
| AQI | 1 |  |  |  |  |  |  |
| LNEC | 0.5931*** | 1 |  |  |  |  |  |
| FDI | -0.0072 | -0.1430** | 1 |  |  |  |  |
| RIS | -0.5290*** | -0.1270** | 0.0406 | 1 |  |  |  |
| GOV | -0.3398*** | -0.5561*** | -0.2311*** | 0.0262 | 1 |  |  |
| LIVING | 0.1411** | 0.3099*** | -0.1285** | 0.0273 | -0.1047* | 1 |  |
| HC | -0.2621*** | -0.1340** | 0.4162*** | 0.3887*** | -0.3422*** | -0.0005 | 1 |
