# Supplementary material for: Evaluation the impact of electricity consumption on China’s air pollution at the provincial level
Source: PLoS One. 2024 Apr 16;19(4):e0301537. doi: 10.1371/journal.pone.0301537 (PMC11020704; doi:10.1371/journal.pone.0301537)
Supplement: S2 File — (DOCX) [file pone.0301537.s002.docx]

**Data Availability Statement**

Sulfur dioxide emissions; Nitrogen oxide emissions; Particulate emissions; Level of industrial structure; Degree of government intervention; Level of living environment; Level of human capital: Data are from the official website of the China Bureau of Statistics at **http://www.stats.gov.cn.**

Carbon dioxide emissions: According to the fourth evaluation report of the IPCC in 2007, the main source of greenhouse gas increase is fossil fuel combustion. Therefore, this article calculates CO2 emissions based on terminal energy consumption data from various provinces over the years. The calculation method is based on the IPCC "Guidelines for National Greenhouse Gas Emissions Inventory" (2006 edition).

Electricity consumption: Data sourced from China Electric Power Statistical Yearbook.

Level of foreign investment: Data sourced from China Statistical Yearbook at **http://www.stats.gov.cn/sj/ndsj/.**
